# Burden of Uterine Fibroids: An African Perspective, A Call for Action and Opportunity for Intervention

**DOI:** 10.18314/cogo.v2i1.1701

**Published:** 2019-08-11

**Authors:** P Igboeli, W Walker, A McHugh, A Sultan, A Al-Hendy

**Affiliations:** 1 University of Nigeria Teaching Hospital, Department of Obstetrics and Gynaecology, Enugu, Nigeria; 2 Department of Obstetrics and Gynecology, Augusta University, Augusta, GA 30912, USA; 3 Department of Obstetrics and Gynecology, University of Illinois, Chicago, Illinois, USA; 4 Xavier University School of Medicine, Oranjestad, Aruba

**Keywords:** Uterine fibroids, Vitamin D, Sub-Saharan Africa

## Abstract

Black women carry the burden of uterine fibroids, (AKA uterine leiomyomas), at a much higher rate than their racial counterparts. Thus, increasing awareness and discovering a solution to an endemic problem that plagues Sub-Saharan Africa is of critical importance, not only for the region itself, but also for the medical community globally. A collaborative, patient oriented, cost effective, and culturally sensitive approach must be at the forefront of this endeavor. While the exact pathogenesis of uterine fibroid development remains elusive, the racial disparity is well documented. Moreover, in the developed world, women are able to seek treatment through surgical and non-surgical means; however, sub-Saharan regions face their own challenges that, if not addressed, can ultimately extinguish the lives of many suffering women. Unfortunately, the literature is scarce on how to prevent fibroid development, which may be critical for women who do not have access to effective interventions. Recent research from our group and others has shown that vitamin D deficiency plays an important role in fibroid development and may be a preventable risk factor. Daily vitamin D supplementation is a low cost, effective intervention that could be implemented throughout the Sub-Saharan region. Similarly, education and increased awareness as to the nature and symptoms of uterine fibroids could improve the quality of life, remove negative social stigma, and reduce morbidity and mortality rates in women who seek medical care with advanced uterine fibroids.

## Introduction

Though the majority of Sub-Saharan African women have minimal formal education, and many do not speak English, the word “fibroid” is woven into their daily conversations - across all cultures and ethnic groups. The mere mention of the word “fibroid” sends chills through their bodies and equates to bad luck for most African women of reproductive age.

Uterine fibroids (UFs), also known as uterine leiomyoma, are the most common benign tumors found in reproductive-aged women of all races and may affect up to 70% of all women by menopause. The highest burden of this condition affects black women, occurring at rates 3–4 times greater in them as compared to their counterparts of other races. It is estimated that 70–80% of black women will harbor fibroids over their lifetime [1–4].

UFs occur in all ages, spanning from menarche to menopause [5,6]. The cause of UFs is largely unknown; however, the racial disparity is well documented [7–10] as well as other risk factors associated with UF development. Risk factors include but are not limited to: age [5–7] as fibroids tend to form as women age, family history [11–13] and low parity [14,15]. Low parity is associated with an increased risk of UFs and it has been hypothesized that uterine involution post-partum serves as the main reason for this protective effect [15]. Furthermore, genetic mutations [16] such as Med-12 have been found in up to 80% of sporadic fibroid lesions. Other DNA polymorphisms have also been associated with fibroid risk. Body weight (increased BMI and/or obesity are associated with an increased risk of UFs) [17,18] and vitamin D deficiency [19–23] has also been associated with increased risk. Recent studies have shown the critical role vitamin D plays in fibroid formation, with individual fibroids expressing lower levels of vitamin D receptors than adjacent healthy tissue, making vitamin D deficiency a crucial, yet preventable risk factor. External use of steroidal hormones especially estrogen and progesterone have also been implicated as risk factors for fibroid development [24,25] as well as early life adverse environmental exposures [26–31].

Of noteworthy mention, although Sub-Saharan Africa has been reported as having the largest population of black people and UFs are highly endemic within this region, almost all past and current core research on the disease has been conducted outside of Sub-Saharan Africa. Additionally, recent studies on the economic impact of UFs have been documented in the United States, while, there is very scarce information as to the economic impact on Sub-Saharan Africa. It is estimated that the annual financial impact of UFs in the United States is 5.9–34.4 billion dollars [32–35]. Moreover, this number may be an underestimation, as at least one-quarter of women reported losing work due to their disease. This is especially important, as though the term ‘fibroid’ is well-known, knowledge as to the causes, prevalence, symptoms, and treatment options are not. In turn, this affects the health care costs and the effectiveness of subsequent treatments. A complete and comprehensive analysis of the direct and indirect costs of UFs in this region is of critical importance.

Some women with fibroids remain asymptomatic and may go through their entire life without knowing they have the disease, especially those with UFs that are small in size and few in number. Some women with fibroids are able to have successful full-term pregnancies and deliver healthy babies without intervention or complications.

For others however, having UFs is a nightmare and oftentimes poses a significant negative impact on their quality of life [36,37]. Due to lack of knowledge, lack of access to care and late presentation, many women in Africa suffer greater morbidity and oftentimes-higher rates of mortality from fibroid disease. Through our experience, many of these women end up at prayer houses or local herbalists who may have good intentions but are untrained in fibroid management. Women die daily due to complications associated with poor disease management.

In the Sub-Saharan African setting and environment, the exhaustive list of barriers and challenges faced by women suffering from UFs all too often include inadequate access to appropriate healthcare facilities, lack of available well-trained providers, poor quality of services when available, affordability issues and poor nutritional status. Major symptoms like menorrhagia, which commonly lead to severe anemia, pelvic pain and pressure, sub-fertility, and miscarriages are but a few examples of the horrific consequences uterine fibroids have on women’s lives [38].

Prolonged delay before presentation for evaluation and management is frequently seen among many Sub-Saharan African women with fibroids [39]. Several factors play a role in the delayed presentation for care including but not limited to: lack of knowledge; corrosive poverty; transportation limitations, procrastination and fear of finding out; misinformation from family and friends, as well as local herbalists and spiritual/prayer houses; wrongful deep-seated cultural beliefs, self-proclaimed spiritual prophets with promises for the cure to fibroids, and fake news among others.

**Figure 1:**
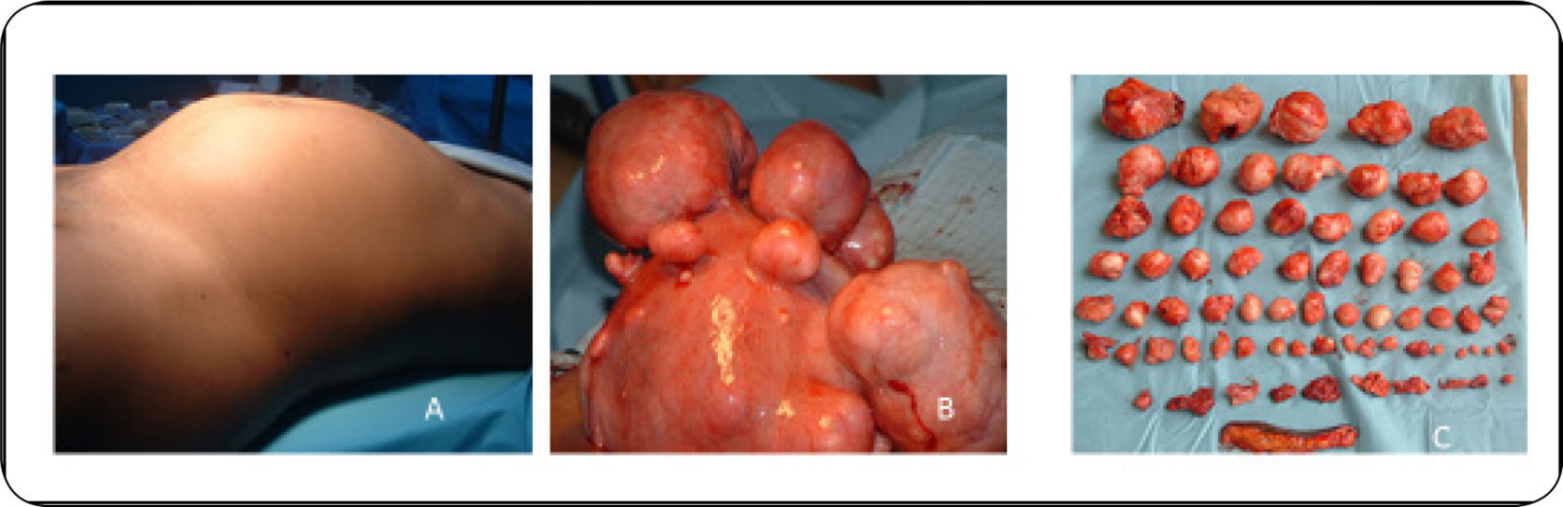
The fibroid tumor burden in Sub-Sahara Africa. (A) Clinical presentation of fibroid at initial evaluation; (B) Uterus with multiple fibroids at open myomectomy; (C) Enucleated fibroid masses following myomectomy. Image courtesy of M&M hospital fibroid images: http://www.mmfertilityhospital.com/

Fibroid symptoms, presentation, diagnosis, and management can span from simple to complex depending on the patient’s state of health, knowledge of fibroids, economic resources, marital status, and available health services within her locality. It is a common occurrence to see black women in Africa presenting with huge fibroids almost appearing as though they are in advanced gestation (Figure 1) when they are not pregnant. They note that their pants and dresses do not fit anymore, and people ask them very embarrassing questions such as “When is the delivery date?” Some cannot get pregnant due to fibroid disease and are labeled by their peers and neighbors as infertile. Many women will not seek medical attention in an effort to hide their disease until marriage due to attached stigma. Some women are driven away, ostracized, abandoned, and divorced due to their inability to conceive or carry a child to full-term.

In managing the burden of fibroid disease among African women, several factors require consideration including, accurate assessment of the epidemiology and burden of disease, availability of services, access to competent healthcare providers, and affordability. Most women live in rural remote areas where medical services and providers are not readily available, or the nearest facility may be quite a distance. Inadequate infrastructure – including poorly designed or maintained roadway systems, lack of transportation, poor communication and power systems, as well as insufficient empowerment for economic development and growth further impede patients’ suffering with fibroid disease from seeking early intervention. Booking structured appointments in most available facilities and hospitals or getting medical attention in a timely fashion is nearly impossible. Not only do these (and others) challenges discourage patients from seeking necessary medical care and treatment, they serve to lure patients into seeking help in places with little to no knowledge and/or expertise in the field of fibroid disease and its management.

Unbeknownst to many Sub-Saharan black women suffering from symptomatic uterine fibroid disease, a clinical diagnosis of the disease is relatively easy to make for a well-trained physician and includes a simple physical examination and/or abdominal or pelvic ultrasonography [40]. Hysterosalpingography, and Hysterosonography are generally available in the diagnosis of uterine and tubal factors of infertility as well as in the detection of submucous fibroids. Other advanced investigative tools such as Magnetic Resonance Imaging (MRI) and Hysteroscopy, are rarely used due to the high cost of service, unavailability of necessary equipment, or properly trained personnel.

Fibroids can be electively and effectively managed when diagnosed early. Some women harboring fibroids do not know they have them and typically go through their entire reproductive life, including pregnancies, without symptoms. Unfortunately, and all too often, African women present very late in the stage of disease when an emergency arises wherein fibroids have grown too large in size and are manifesting with debilitating health outcomes such as severe hemorrhage and grave anemia—both of which can lead to increased morbidity and sometimes mortality, - particularly under already compromised conditions frequently seen in populations with little to no resources. Management of patients under these conditions can become complex. For those patients who are stable but symptomatic, medical interventions may be initiated primarily to provide symptomatic relief from excessive bleeding, correct anemia, and slow further fibroid growth and decrease pelvic pain. Administering interventional drugs is yet another method utilized in treating UFs and typically includes the use of combined oral contraceptives, progesterone-releasing intrauterine devices, progestin-only preparations, androgens, gonadotropin releasing hormone analogues and both estrogen and progesterone receptor antagonists and mixed receptor antagonist/agonists [41]. However, these options usually fail to provide continued efficacy and relief after cessation of medication(s), which may lead to symptom recurrence. Many patients are then left to seek more invasive interventions as their only option.

In developed countries with advanced medical facilities such as the US, Western Europe, and Japan, patients are presented with an array of surgical options in the management of fibroid disease, depending on the severity of the patient’s tumor and/or burden of disease. Surgical treatment options may include myomectomy and hysterectomy (open versus minimally invasive approach), uterine artery embolization (UAE), Magnetic Resonance guided Focused Ultrasound (MRgFUS), and Hysteroscopic myomectomy (for intra-cavity fibroids) among others.

Furthermore, most fibroid surgical procedures are readily available and are carried out under heavily regulated conditions by qualified surgeons and blood bank services to optimize patient safety and procedural outcomes. This is typically not the case in the African Sub-Saharan region where there is limited access to quality hospitals with regulated standards of care and highly skilled experts/surgeons, and inadequate laboratory and blood products. For example, in the USA, hysterectomy is the preferred treatment for symptomatic fibroids but in Africa, it is the least accepted by patients due to cultural norms and beliefs. Most women would prefer to die and be buried with their uteri because their culture holds the belief that they need their womb for childbearing in the next life. In Sub-Saharan Africa, most treatments for symptomatic UFs involve myomectomy, a procedure that is typically done under subpar conditions that compromises patient safety and results in poor health outcomes. Most morbidity and mortality arise from less-than-optimal pre-operative assessments and patient stabilization, intra-operative complications that center on anesthesia, excessive blood loss, surgical injury to adjacent organs particularly bowels where the surgeon encounters dense pelvic adhesion(s), prolonged operating time, lack of available inter-specialty consultations, lack of adequate infrastructure such as steady electricity and functional operating rooms and equipment. Post operatively, complications may include hemorrhage, shock, fever, wound infection, and subsequent dehiscence culminating in protracted hospitalizations [42]. Far-reaching consequences include destruction of endometrium during fibroid enucleation especially when UFs are multiple in number and bulky - causing amenorrhea and iatrogenic infertility in reproductive-aged women. The physical and psychological trauma inflicted on a family unit because of infertility can result in dissolution of the marriage. For these reasons and more, African women prefer other less invasive options rather than surgery.

Interestingly, some novel medications are currently being investigated as potential oral alternatives to surgery in the management of UFs such as selective progesterone receptor modulators and oral nonpeptide gonadotrophin releasing hormone antagonists [43,44]. Most of these interventions are currently far too expensive for women residing in poorly resourced economies like that of Sub-Saharan Africa. Furthermore, the periodic patient follow-up and rigid compliance regimens that these interventions may require make them less than optimal options due to lack of availability and affordability in this region.

In the management of African women with fibroids, cost containment is of pivotal importance. Therefore, considerable thought must be given to the development of low-cost interventions that achieve an exceedingly high rate of compliance. Most Sub-Saharan African women do not carry health insurance to cover the expense of fibroid disease management and/or treatment; nor do they have access to regular care, advanced interventions or access to vitamin enriched nutritional supplements – like their counterparts found in more developed countries.

Recent studies from our group Al-Hendy et. al. [19–22,39,45–51] and others [23,52–54] have shown that vitamin D deficiency is an important risk factor in the development of UFs. Not surprisingly, vitamin D deficiency is also highly prevalent among black populations. Higher melanin concentrations in black populations decrease the absorption of ultraviolet rays from the sun which leads to decreased vitamin D synthesis and production. Additionally, increased incidence of lactose intolerance in this same population leads to decreased milk consumption, in turn, exacerbating the deleterious effects of vitamin D deficiency. Recent studies have shown that individual fibroids express lower levels of vitamin D receptors in comparison to surrounding healthy myometrial tissue. These findings are concerning and may shed light on the higher incidence of uterine fibroids in black women. Further demonstration of an actual reduction in the formation of new fibroids and further growth retardation in existing fibroids when appropriate doses of vitamin D are administered [55]. Daily vitamin D3 supplementation is a low-cost intervention that can potentially help reduce the burden of uterine fibroid disease in low resource economies like that of Sub-Saharan African. The use of vitamin D supplementation in women with early and less severe uterine fibroid disease as well as in pre-symptomatic women from menarche to age 30 when fibroid disease is less likely to produce symptoms or has not yet fully manifested appears as a window of hope and opportunity for reproductive age Sub-Saharan African women [55]. Vitamin D is a readily available and affordable supplement. Importantly, by educating women about UFs and by encouraging early evaluation for diagnosis, timely interventions can be introduced to reduce rates of morbidity and mortality seen in patients who present late with advanced tumor growth.

Though Sub-Saharan women bear the greatest brunt of this global disease, the African environment has not yet been seriously studied to comparatively determine whether environmental impact plays a role in fibroid formation.

It is rather disturbing that very little attention has been given to conducting research for more practical methods in disease diagnosis, management, and treatment designed to meet the unique needs and challenges faced by Sub-Saharan African women, particularly when they bear such significant burden of uterine fibroid disease. Little to no data appears to be hailing from the Sub-Saharan region; however, perhaps international collaborations and support could ameliorate this effort if further championed by the international community for the benefit of all sides [56–60].

Finally, promising opportunities await through international collaborative researchers who could begin to explore and generate the much-needed global comparative data on the environmental impact as well as comparative effectiveness of the varying treatment modalities in Sub-Saharan Africa. Collaborative ventures such as those described above will be of mutual benefit to all parties involved including Sub-Saharan Africa and the rest of the world. Furthermore, such studies would serve as models for planning and developing less expensive, affordable and less invasive modalities of care; thereby reducing the colossal health care expenditure associated with UFs, but most importantly, it will have a significant positive impact on health outcomes as well as the quality of life for all women during their reproductive years, worldwide.
